# Palaeoproteomic investigation of an ancient human skeleton with abnormal deposition of dental calculus

**DOI:** 10.1038/s41598-024-55779-y

**Published:** 2024-03-11

**Authors:** Yoko Uchida-Fukuhara, Shigeru Shimamura, Rikai Sawafuji, Takumi Nishiuchi, Minoru Yoneda, Hajime Ishida, Hirofumi Matsumura, Takumi Tsutaya

**Affiliations:** 1 Department of Oral Morphology, Faculty of Medicine, Dentistry and Pharmaceutical Sciences, Okayama University, Okayama, 700-8525 Japan; 2 Research Center for Integrative Evolutionary Science, The Graduate University for Advanced Studies (SOKENDAI), Kanagawa, 240-0193 Japan; 3 Institute for Extra-Cutting-Edge Science and Technology Avant-Garde Research (X-STAR), Japan Agency for Marine-Earth Science and Technology (JAMSTEC), Yokosuka, 237-0061 Japan; 4 Department of Human Biology and Anatomy, Graduate School of Medicine, University of the Ryukyus, Okinawa, 903-0215 Japan; 5 Research Center for Experimental Modeling of Human Disease, Kanazawa University, Kanazawa, 920-8640 Japan; 6 The University Museum, The University of Tokyo, Tokyo, 113-0033 Japan; 7 Mt. Olive Hospital, Okinawa, 903-0804 Japan; 8 School of Health Sciences, Sapporo Medical University, Hokkaido, 060-8556 Japan; 9 Biogeochemistry Research Center (BGC), Japan Agency for Marine-Earth Science and Technology (JAMSTEC), Yokosuka, 237-0061 Japan

**Keywords:** Biological anthropology, Gum disease

## Abstract

Detailed investigation of extremely severe pathological conditions in ancient human skeletons is important as it could shed light on the breadth of potential interactions between humans and disease etiologies in the past. Here, we applied palaeoproteomics to investigate an ancient human skeletal individual with severe oral pathology, focusing our research on bacterial pathogenic factors and host defense response. This female skeleton, from the Okhotsk period (i.e., fifth to thirteenth century) of Northern Japan, poses relevant amounts of abnormal dental calculus deposition and exhibits oral dysfunction due to severe periodontal disease. A shotgun mass-spectrometry analysis identified 81 human proteins and 15 bacterial proteins from the calculus of the subject. We identified two pathogenic or bioinvasive proteins originating from two of the three “red complex” bacteria, the core species associated with severe periodontal disease in modern humans, as well as two additional bioinvasive proteins of periodontal-associated bacteria. Moreover, we discovered defense response system-associated human proteins, although their proportion was mostly similar to those reported in ancient and modern human individuals with lower calculus deposition. These results suggest that the bacterial etiology was similar and the host defense response was not necessarily more intense in ancient individuals with significant amounts of abnormal dental calculus deposition.

## Introduction

Ancient human skeletons sometimes show abnormal and extremely severe pathological conditions that could be rarely observed in modern human populations^1,2^. These extreme cases emphasize both human resilience and vulnerability to disease in the absence of modern healthcare interventions^3,4^. Humans and pathogens coevolved and various ancient pathogens are not equivalent to their contemporary descendants^5,6^. Ancient severe pathological conditions that cannot be seen today could have existed due to the lack of modern medical interventions or different bacterial etiologies. Detailed investigation of these extreme cases would be important as they shed light on the breadth of potential interactions between humans and diseases, and reveal differences between past disease etiologies and present-day pathogens.

In this study, we used palaeoproteomics to investigate the etiology of and host resilience to periodontal disease in an ancient human skeleton showing abnormal deposition of dental calculus with severe periodontal disease. Dental calculus is a calcified oral plaque that promotes periodontal disease^7^ and is habitually removed in modern dental care. In contrast, abnormal depositions of dental calculus, where a large calculus deposition entirely covers the occlusal surface of at least one tooth, could be occasionally observed in ancient human skeletons. Such examples include a late Saxon skeleton from Nottinghamshire, UK^8^, a skeletal individual from eighth to tenth centuries Clarensac cemetery from Nîmes, France^9^, and the subject of this study, an Okhotsk skeleton from Hokkaido, Japan^10^ (Supplementary Text S1). Dental calculus entraps and preserves microparticles, DNA, and proteins originating from the environment, host, microbiome, and diet. Therefore, dental calculus provides molecular clues to help understand the lifeways of the host, pathological conditions, and disease etiology in the past^11,12^. Analyzing abnormally deposited dental calculus can further reveal the pathogenic cause of oral pathology and the defense response of the host.

Palaeoproteomics of dental calculus, applied in this study, is an effective method for investigating both the etiology of and host responses to ancient periodontal disease^13–15^. Proteins are functional agent, and their expression differs in response to pathological conditions. These pieces of evidence, revealing information on functional oral pathologic processes, could not be obtained solely by DNA analysis, which could only reveal the presence of certain taxa in analyzed specimens. The paleoproteomic analytic potential of dental calculus for studying health and diseases in the past has not been fully exploited (however, see references^13–16^) despite successful applications in studies aiming at dietary reconstruction^17–22^.

By applying palaeoproteomics to abnormally deposited dental calculus from a skeletal individual with severe periodontal disease, we aimed at answering (i) whether the pathogenic factors associated with the severe periodontal disease in this individual differed from modern and ancient human individuals with lower calculus deposition, and (ii) to what extent the extreme oral pathological conditions caused pathological stress to the host.

### The subject individual, HM2-HA-3

**Figure 1:**
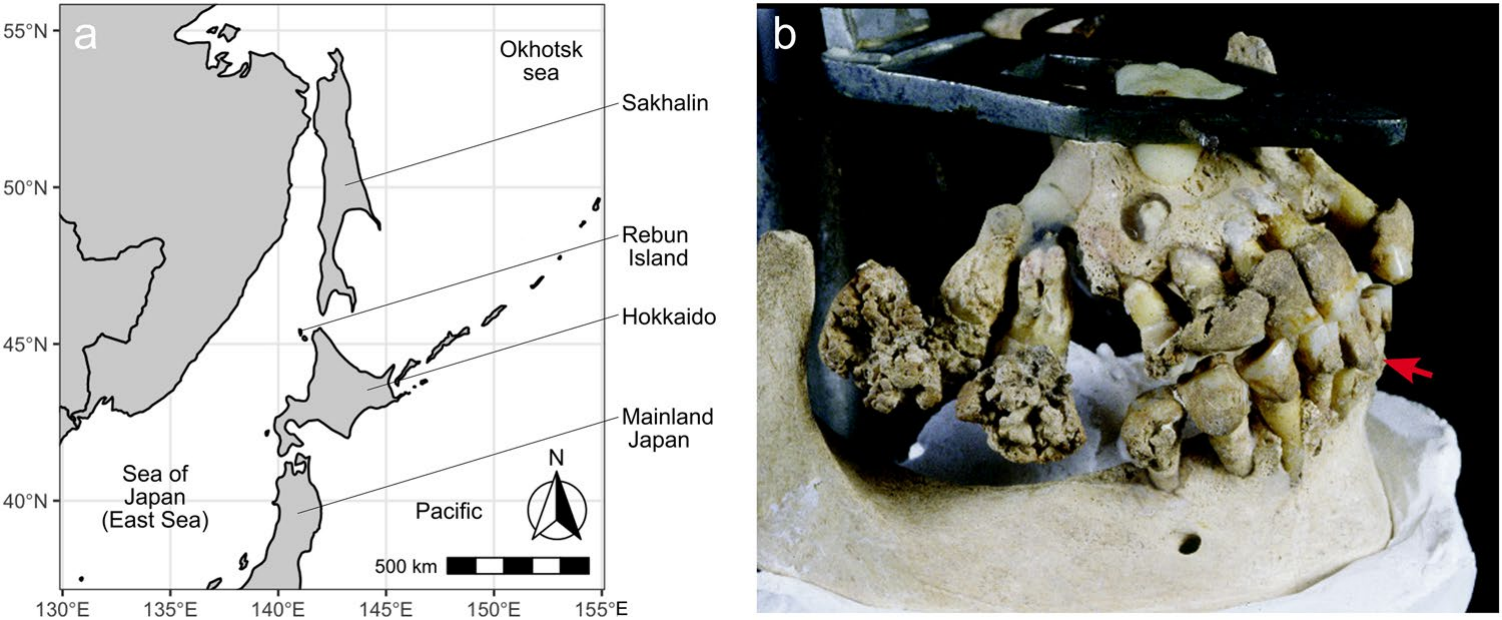
(**a**) Map of Rebun Island. The map was drawn with the packages ggplot2 (version 3.4.0) and rnaturalearth (version 0.3.2) on version 4.2.2 of R. (**b**) Right buccal aspect of the HM2-HA-3 maxilla and mandible. A red arrow indicates the sampled calculus (i.e., from the lower right permanent first incisor).

HM2-HA-3 is a female skeleton, aged 34–54 years at death, excavated in 1992 from the Hamanaka 2 site (Fig. 1) on Rebun Island, Hokkaido, Japan^23^. The most notable feature of this individual is the abnormal deposition of large amounts of dental calculus (Fig. 1^10^). The morphological characteristics of this individual have been previously described in detail^10^. Briefly, most skeletal elements of HM2-HA-3 were missing; only a part of the cranium, an upper limb, and trunk bones were present, though the mandible and maxilla, including erupted teeth were well-preserved. Heavy deposits of dental calculus were present, especially on the right side of the dentition. These calculus deposits are predominantly located above the cementoenamel junction, a feature of supragingival calculus. These deposits were primarily found on the right upper second and third molars (Fig. 1). The occlusal surfaces of these molars are completely covered by calculus deposits and present a non-smooth surface.

HM2-HA-3 also exhibits extreme oral pathological conditions. Caries are not present in any of the remaining teeth but HM2-HA-3 presents apical lesions with cementum hyperplasia, rounded cavities in the root apex, and severe periodontal disease including resorption of the alveolar process^10^. Periodontitis-related horizontal alveolar bone resorption was prominent in HM2-HA-3, and the mandibular right molars had been completely lost with severe resorption of the crest. This individual would likely have suffered from the periodontal disease since the relatively early stages of her life, when the right side of her jaws would have become almost completely unusable for masticatory function^10^. As a result, HM2-HA-3 showed severe tooth wear on her left teeth, which were not covered by calculus. Furthermore, alveolar bone resorption at the root branch was observed on the upper right side, suggesting the presence of endodontic-periodontal disease. Abnormal calculus deposition would have facilitated periodontal tissue collapse in the same region. Taken together, these conditions show that normal masticatory function would have been impaired in this individual.

HM2-HA-3 was found in an archaeological site belonging to the Okhotsk culture. The Okhotsk culture was distributed along southern Sakhalin Island, the northeastern coast of Hokkaido, and the Kuril Islands during the fifth to thirteenth centuries^24^. The Okhotsk people predominantly subsisted on fishing, and it is estimated that marine foods comprised more than 80% of their dietary protein intake^25,26^. Although a few crop remains have been excavated from Okhotsk sites^27^, it is believed that plant horticulture was not practiced in the Okhotsk culture^24^. Because of their low carbohydrate intake, the caries rate of Okhotsk people was remarkably lower than in Jomon hunter-gatherers^28^. Physical anthropological measures of oral health, such as the frequency of linear enamel hypoplasia, in the Okhotsk people were generally better than in the Jomon hunter-gatherers of mainland Japan^29^. Even though, no other Okhotsk human skeletons show such an abnormal calculus deposition seen in HM2-HA-3^10^. The archaeological and anthropological contexts of the Okhotsk culture are summarized in the Supplementary Text S2.

## Results

### Chronological age and diet

Elemental and isotopic results of the rib bone collagen sample from HM2-HA-3 are shown in Table 1. Bone collagen extracted from the rib of HM2-HA-3 showed acceptable %C (44.5%), %N (16.4%), and C/N ratio (3.17)^30,31^, suggesting good molecular preservation of this individual.

**Table 1:** Results of stable isotope analysis and radiocarbon measurement.

ID	sex	Age (y)	Element	%C	%N	δ^13^C	δ^15^N	C/N	^14^C age (BP)	Reference
1480	M	40–50	Skull	43.9	15.1	-13.2	19.0	3.4	–	Naito et al., 2010^25^
1496	F	30–40	Skull	43.8	15.7	-12.9	18.6	3.3	–	Naito et al., 2010^25^
NAT002	F	40–49	–	41.8	15.0	-12.9	19.3	3.2	–	Okamoto et al., 2016^35^
HM2-HA-3	F	35–54	Rib	44.5	16.4	-13.0	19.3	3.2	1777 ± 37	This study

Previously reported data from other skeletal individuals from the Hamanaka 2 site are also shown.

**Figure 2:**
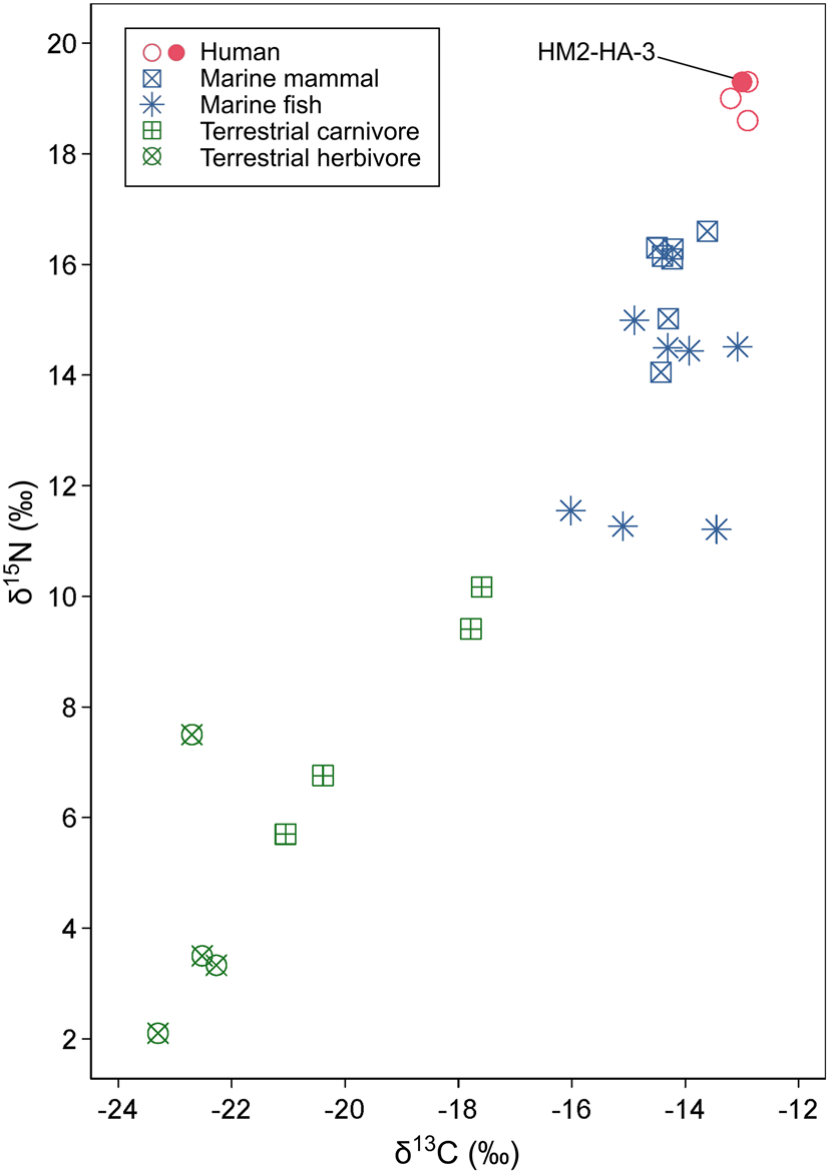
Carbon and nitrogen stable isotopic results of faunal and human bone collagen.

The calibrated radiocarbon age of HM2-HA-3 was 485–760 cal AD with 95.4% posterior probability and 565–678 cal AD with 68.3% posterior probability. Considering the chronology of the Hamanaka 2 site^32^, this age falls in the earlier Okhotsk period. The δ^13^C and δ^15^N values of bone collagen from HM2-HA-3, which mostly represent protein dietary components assimilated during ~10 years before death^33,34^, were -13.0‰ and 19.3‰, respectively. These isotope ratios are shown in Fig. 2 along with the previously reported values from other human skeletons excavated at the Hamanaka 2 site^25,35^ and faunal bones excavated from another Okhotsk site (Moyoro site^26^). These comparisons showed that most dietary proteins of HM2-HA-3 were obtained from marine foods and there were no apparent differences in dietary food sources between HM2-HA-3 and other Okhotsk individuals excavated from the Hamanaka 2 site (Fig. 2).

### Dental calculus proteome

We identified a total of 96 protein groups from the dental calculus of HM2-HA-3, excluding keratins and common laboratory contaminants. Of these, 81 and 15 protein groups originated from humans (Table 2) and bacteria (Table 3), respectively. The calculus displayed a high (i.e., 92.1%) OSSD score, suggesting good protein preservation^20^. The peptide deamidation rates, the approximate proxy for ancient protein authenticity^36,37^, derived from the four fractions ranging between 38.7–54.8% and 30.7–37.7% for asparagine and glutamine in human proteins, respectively (Supplementary Table S1). As the deamidation rate of modern proteins is typically below 20%, the human proteins identified in the dental calculus of HM2-HA-3 would originate from ancient times^13^. In contrast, bacterial proteins showed lower asparagine and glutamine deamidation rates (4.9–23.2% and 4.2–24.0%, respectively) (Supplementary Table S1). The number of asparagine and glutamine residues in the identified bacterial proteins was below 8, the precise deamidation rates could thus not be calculated.

**Table 2:** Human protein groups identified in the dental calculus of HM2-HA-3.

Protein ID	Protein name	Gene name	N. of razor + unique peptides					Sequence coverage (%)	Score
			Total	SP3-S	UF-S	SP3-P	UF-P		
E7EQB2	Lactotransferrin (fragment)	LTF	22	12	11	16	13	40.7	323.31
P01024	Complement C3	C3	21	11	11	15	11	17.2	323.31
P05164-2	Isoform H14 of myeloperoxidase	MPO	20	8	9	16	11	39.8	202.58
P01023	Alpha-2-macroglobulin	A2M	16	10	8	8	6	16.1	129.04
A0A024R6I7	Alpha-1-antitrypsin	SERPINA1	14	7	9	6	7	44.3	287.77
P30740	Leukocyte elastase inhibitor	SERPINB1	14	6	8	3	5	44.9	11.99
P12273	Prolactin-inducible protein	PIP	10	6	8	6	9	73.3	323.31
P01008	Antithrombin-III	SERPINC1	10	5	7	6	6	35.1	65.43
P01871	Immunoglobulin heavy constant mu	IGHM	9	1	3	2	8	28.3	23.09
P05109	Protein S100-A8	S100A8	9	7	6	5	6	81.7	113.57
P06702	Protein S100-A9	S100A9	9	6	7	7	8	60.5	323.31
Q6P5S2	Protein LEG1 homolog	LEG1	8	6	6	5	8	42.7	157.65
P00450	Ceruloplasmin	CP	7	2	5	3	4	9.0	39.11
P01036	Cystatin-S	CST4	6	3	3	3	1	53.2	9.91
P68871	Hemoglobin subunit beta	HBB	6	3	4	3	6	53.7	131.40
J3QLC9	Haptoglobin (fragment)	HP	6	2	2	3	4	19.2	12.79
P01011	Alpha-1-antichymotrypsin	SERPINA3	6	4	4	3	3	20.3	323.31
A0A8V8TL71	Actinin alpha 4	ACTN4	5	1	3	2	1	7.4	80.754
P0DUB6	Alpha-amylase 1A	AMY1A	5	1	1	3	4	11.7	2.7398
P07237	Protein disulfide-isomerase	P4HB	5	0	4	1	0	11	1.5164
P01833	Polymeric immunoglobulin receptor	PIGR	5	3	1	2	3	10.3	9.1261
A0A7P0Z497	Peptidyl–prolyl *cis–trans* isomerase	PPIB	5	3	2	1	1	27.1	8.4126
P29508-2	Isoform 2 of Serpin B3	SERPINB3	5	5	4	1	3	22.2	266.53
A0A0C4DGN4	Zymogen granule protein 16B	ZG16B	5	5	3	4	3	37.2	192.94
P25311	Zinc-alpha-2-glycoprotein	AZGP1	4	2	3	3	2	19.1	4.6576
Q8N4F0	BPI fold-containing family B member 2	BPIFB2	4	2	1	3	1	13.3	1.2089
P08246	Neutrophil elastase	ELANE	4	0	2	2	3	31.1	23.103
A0A286YEY1	Immunoglobulin heavy constant alpha 1 (fragment)	IGHA1	4	1	3	2	4	16.3	68.605
P01877	Immunoglobulin heavy constant alpha 2	IGHA2	4	3	1	2	2	21.8	16.597
P01591	Immunoglobulin J chain	JCHAIN	4	1	4	1	1	27.7	1.873
P61626	Lysozyme C	LYZ	4	4	3	3	2	41.2	38.618
Q9HD89	Resistin	RETN	4	1	3	2	4	51.9	323.31
Q09666	Neuroblast differentiation-associated protein AHNAK	AHNAK	3	0	1	0	2	1.3	0.38438
A0A590UJZ9	Deleted in malignant brain tumors 1 protein	DMBT1	3	1	1	3	2	8.2	78.756
P31025	Lipocalin-1	LCN1	3	2	3	1	0	14.2	2.7264
A0A8V8TKR9	Moesin	MSN	3	1	0	2	0	5.7	0.34829
Q14686	Nuclear receptor coactivator 6	NCOA6	3	2	0	0	1	1.5	0.16146
P12724	Eosinophil cationic protein	RNASE3	3	1	0	3	2	25	194.29
A0A494C0J7	Transglutaminase-like domain-containing protein	–	3	2	0	1	1	5.3	77.907
A0A2R8YH90	Tropomyosin 4	TPM4	3	2	2	1	0	10.9	2.2422
A0A0A0MTS7	Titin	TTN	3	1	1	0	1	0.1	0.79787
Q9HCE9	Anoctamin-8	ANO8	2	1	1	1	1	1.9	0.35178
P04083	Annexin A1	ANXA1	2	1	2	0	0	6.9	2.0362
P02743	Serum amyloid P-component	APCS	2	0	0	0	2	9.4	0.46515
P20160	Azurocidin	AZU1	2	1	1	1	2	9.2	7.0993
A0A8V8TLP6	Complement C4A (Rodgers blood group)	C4A	2	1	0	1	2	2	14.849
A5YKK6-4	Isoform 4 of CCR4-NOT transcription complex subunit 1	CNOT1	2	1	1	1	0	1.7	1.6788
K7ESB6	Casein kinase 1 gamma 2 (fragment)	CSNK1G2	2	0	0	1	1	12.7	1.1282
P59666	Neutrophil defensin 3	DEFA3	2	1	0	1	1	19.1	46.255
Q8TB45	DEP domain-containing mTOR-interacting protein	DEPTOR	2	0	2	0	0	6.4	0.183
Q96M86	Dynein heavy chain domain-containing protein 1	DNHD1	2	1	1	0	0	0.6	0.46915
C9JYU7	Mitotic deacetylase associated SANT domain protein (fragment)	MIDEAS	2	0	0	0	2	43.1	0.32619
E7EUT5	Glyceraldehyde-3-phosphate dehydrogenase	GAPDH	2	0	0	1	1	10.8	2.864
Q92820	Gamma-glutamyl hydrolase	GGH	2	1	1	1	0	8.5	1.4863
P62805	Histone H4	H4C16	2	1	0	1	0	17.5	0.019644
A0A0G2JIW1	Heat shock 70 kDa protein 1B	HSPA1B	2	0	1	0	1	4.8	0.55721
A0A7P0TAI0	78 kDa glucose-regulated protein	HSPA5	2	1	0	1	1	4.2	0.66496
P01834	Immunoglobulin kappa constant	IGKC	2	2	2	2	1	31.8	30.836
P0DOY3	Immunoglobulin lambda constant 3	IGLC3	2	0	1	1	1	33	9.558
A0A3B3IU98	IQ motif and Sec7 domain ArfGEF 1 (fragment)	IQSEC1	2	1	0	0	2	3.2	1.2623
P06870-2	Isoform 2 of Kallikrein-1	KLK1	2	2	2	0	1	12.5	2.8386
P13796	Plastin-2	LCP1	2	1	1	0	0	3.2	0.11803
Q8IWC1-4	Isoform 4 of MAP7 domain-containing protein 3	MAP7D3	2	0	1	1	0	3.7	0.38741
P98088	Mucin-5AC	MUC5AC	2	0	2	0	0	0.3	0.06592
Q9UKX3	Myosin-13	MYH13	2	0	0	0	2	2.3	0.24148
Q7Z406-5	Isoform 5 of Myosin-14	MYH14	2	0	1	1	1	1.6	1.6056
A0A0A0MRM2	Nebulin related anchoring protein	NRAP	2	0	0	1	1	1.4	0.26403
Q7Z2Y5-2	Isoform 2 of Nik-related protein kinase	NRK	2	0	0	2	0	1.9	0.16886
Q13310-3	Isoform 3 of Polyadenylate-binding protein 4	PABPC4	2	0	0	0	2	5	0.41886
O75594	Peptidoglycan recognition protein 1	PGLYRP1	2	1	2	1	2	19.9	17.412
P24158	Myeloblastin	PRTN3	2	1	0	1	1	8.2	0.45503
A0A7I2V2H3	Proteasome subunit alpha type	–	2	0	0	2	0	15.4	3.0049
P28065	Proteasome subunit beta type-9	PSMB9	2	0	0	1	2	11.4	1.1251
Q5VT52-2	Isoform 2 of regulation of nuclear pre-mRNA domain-containing protein 2	RPRD2	2	0	0	1	1	1.8	0.30317
P25815	Protein S100-P	S100P	2	1	2	1	2	30.5	25.995
F8W0Q0	Sodium voltage-gated channel alpha subunit 8 (fragment)	SCN8A	2	0	2	0	0	3.1	0.26391
P48595	Serpin B10	SERPINB10	2	0	1	0	1	5	0.88109
A0A087WUD9	Serpin family G member 1	SERPING1	2	2	1	0	0	6.2	0.53009
P02814	Submaxillary gland androgen-regulated protein 3B	SMR3B	2	1	1	1	1	65.8	2.6144
P50552	Vasodilator-stimulated phosphoprotein	VASP	2	0	2	0	1	6.6	1.8437
Q6N043-2	Isoform 2 of zinc finger protein 280D	ZNF280D	2	0	1	0	1	3.6	0.059397

**Table 3:** Oral bacterial protein groups identified in the dental calculus of HM2-HA-3.

Protein ID	Protein name	Taxonomy	Strain	Periodontal	N. of razor + unique peptides					Sequence coverage (%)	Score	Note
					Total	SP3-S	UF-S	SP3-P	UF-P			
SEQF2705_00640	Inosamine-phosphate amidinotransferase 1	*Actinomyces* *israelii*	DSM 43320	Yes	8	2	1	2	7	32.0	36.74	
SEQF3180_02237	Enolase	*Actinomyces* *sp.HMT* *169*	F0496		7	1	2	3	7	22.0	68.84	
SEQF1598_00449	Flagellar filament 33 kDa core protein	*Selenomonas* *sputigena*	ATCC 35185	Yes	3	1	0	0	3	10.8	12.62	
SEQF1674_00209	Flagellin	*Fretibacterium* *fastidiosum*	SGP1	Yes	3	0	0	2	1	5.5	12.82	
SEQF1013_00339	Enolase	*Corynebacterium* *matruchotii*	ATCC 14266		2	0	1	0	1	9.9	11.596	calcifying bacterium
SEQF1604_01614	Major outer membrane protein P.IB	*Cardiobacterium* *hominis*	ATCC 15826		2	0	0	2	0	8.8	26.671	
SEQF2454_00889	Flagellar filament 31 kDa core protein	*Selenomonas* *sp.* *HMT* *892*	F0426		2	0	0	2	0	9.8	8.7738	
SEQF3095_01402	Inosamine-phosphate amidinotransferase 1	*Actinomyces* *sp.* *HMT* *171*	F0337		2	0	0	1	2	16.8	14.42	
SEQF2745_00190	18 kDa heat shock protein	*Actinomyces* *sp.* *HMT* *414*	F0588		2	0	1	0	1	23.8	8.886	
SEQF2434_01156	Fumarate reductase flavoprotein subunit	*Campylobacter* *gracilis*	RM3268		2	1	0	0	1	5.2	9.5569	
SEQF1871_01017	Flagellar filament 33 kDa core protein	*Treponema* *denticola*	US-Trep/F0459	Yes	2	0	0	0	2	16.8	8.9183	
SEQF3226_01738	Minor fimbrium subunit Mfa1	*Porphyromonas* *gingivalis*	AFR5B1	Yes	2	2	1	0	1	7	9.9852	
SEQF2745_00395	Fimbrial subunit type 1	*Actinomyces* *sp.* *HMT* *414*	F0588		2	0	1	0	2	6	58.435	
SEQF2743_01115	Lys-gingipain W83	*Porphyromonas* *gingivalis*	W50	Yes	2	0	1	1	0	3.9	35.543	
SEQF3145_00537	1,4-alpha-glucan branching enzyme GlgB	*Actinomyces* *oricola*	R5292	Yes	2	0	0	1	1	4.9	9.6191	

The identified human proteins were classified with GO term using the PANTHER software^38^. Among the assigned protein class, 13.9% represented the “defense/immunity.” Among the proteins categorized in this class, peptidoglycan recognition protein 1 was one of the innate immune system proteins and functions to directly kill bacteria by recognizing and cleaving peptidoglycans on the bacterial wall^39^. Neutrophil elastase is among the antimicrobial peptides abundant in the saliva and gingival crevicular fluid in the oral cavity and is involved in local defense mechanisms^40^.

We identified a total of 15 proteins from 13 bacterial taxa from the calculus. Eight of these originated from six bacterial taxa that are reportedly associated with periodontal disease in modern patients (Table 3). We identified two of the three “red complex” bacteria, the most notable core bacterial species in the severe form of periodontal disease (*Porphyromonas* *gingivalis* and *Treponema* *denticola*). In addition, among the identified bacterial taxa, *Selenomonas* *sputigena* and *Fretibacterium* *fastidiosum* are reportedly associated with severe periodontal disease in modern humans^41,42^, while *Actinomyces* *dentalis* and *Actinomyces* *israelii* were identified in patients with severe periodontal disease^43^. Pathogenic factors of *P.* *gingivalis*, a proteolytic enzyme of Lys-gingipain W83 and Mfa1 were identified in the calculus with well-annotated MS2 spectra (Supplementary Figure S2)^44^. Moreover, pathologically invasive proteins, such as *T.* *denticola* flagellar filament 33-kDa core protein, *F.* *fastidiosum* flagellin, and *S.* *sputigena* flagellar filament 33-kDa core protein, were also identified with well-annotated MS2 spectra (Supplementary Figure S2). These flagellar proteins are associated with bacterial motility and could initiate immune responses by interacting with toll-like receptor 5 in the host^45–48^. We could not identify any bacterial taxa and dental caries-associated proteins. Our BLAST search indicated that the peptide sequences of these periodontal disease-associated bacterial proteins only occur in certain bacterial genera (Supplementary Table S2).

**Figure 3:**
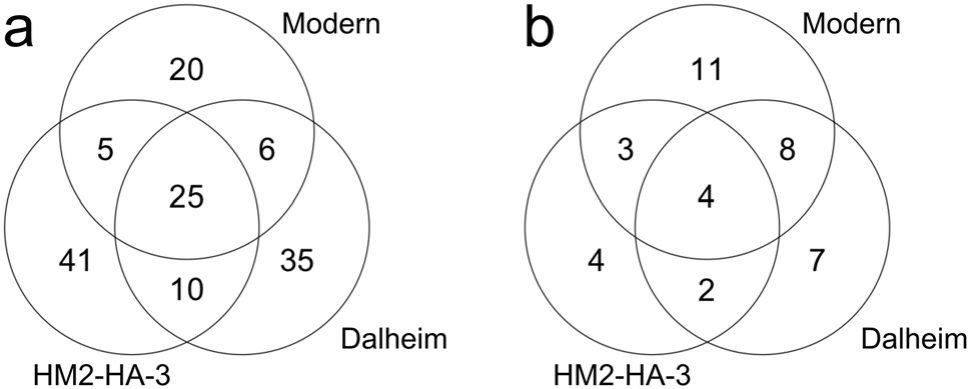
Venn diagrams of (**a**) human proteins and (**b**) bacterial taxa identified in the ancient dental calculus of HM2-HA-3 (this study) as well as in the dental calculus samples from medieval Dalheim and modern patients^12^.

We compared the protein groups or bacterial taxa identified in the dental calculus of HM2-HA-3 with those identified in a previous palaeoproteomic analysis of ancient human dental calculus from medieval Dalheim, Germany as well as those of modern European patients with periodontitis and dental caries^13^. As presented in Fig. 3, 49.4% (40/81) of the human proteins and 69.2% (9/15) of the bacterial taxa identified in HM2-HA-3 calculus were also identified either in Dalheim or modern calculus^13^. with the common bacterial taxa being *P.* *gingivalis*, *A.* *israelii,* *Actinomyces* *sp.* HMT 414, and *Corynebacterium* *matruchotii*. Bacterial species unique to HM2-HA-3 included *S.* *sputigena,* *Actinomyces* *sp.* HMT 169, *Selenomonas* *sp.* HMT 892, and *Campylobacter* *gracilis*^49^.

The “defense/immunity” protein class proportion calculated by PANTHER was similar between the Dalheim (10.4%) and HM2-HA-3 (13.9%) calculi while that in modern calculus was higher (20.8%). The proportion of “immune system”-assigned biological processes calculated by PANTHER was lower in HM2-HA-3 (6.9%) than in Dalheim (8.1%) and modern (10.8%) dental calculi.

Finally, we performed a proteomic analysis of a rib bone sample of HM2-HA-3 to investigate the potential presence of systematic diseases. We identified a total of 59 human proteins, most of them being bone proteins (Supplementary Table S3). We could not identify any systematic disease-associated protein.

## Discussion

The palaeoproteomic analysis of abnormally deposited dental calculus conducted here provided molecular insights into the pathological conditions of the oral cavity of HM2-HA-3. We identified both pathogenic factors and bioinvasive proteins (i.e., Lys-gingipain W83, Mfa1, flagellin, and flagellar filament 33-kDa) from bacterial taxa reportedly associated with periodontal disease in modern patients. The identification of these proteins provides molecular support for the periodontal disease of this individual originally diagnosed based solely on physical characteristics. These bacterial proteins are associated with periodontal disease pathogenesis and development as well as with the secretion of inflammatory cytokines^45–48,50^.

Of the 13 bacterial taxa identified from the calculus of HM2-HA-3, seven (53.8%) are reportedly associated with periodontal disease in modern clinical medicine (Table 3), in particular, two of the three red complex bacterial taxa. Proteins from the red complex bacteria have frequently been identified in both modern and ancient human dental calculus samples^13,15,51,52^. In this study, the pathogenic protein of *P.* *gingivalis* and bioinvasive protein of *T.* *denticola* were confidently identified^53^, providing direct evidence of red complex bacterial involvement in periodontal disease etiology. Although the involvement of the remaining seven bacterial taxa in the etiology of periodontal disease remains unclear, our results confidently indicate that periodontal disease bacterial etiology in HM2-HA-3 was similar to that in modern patients.

**Figure 4:**
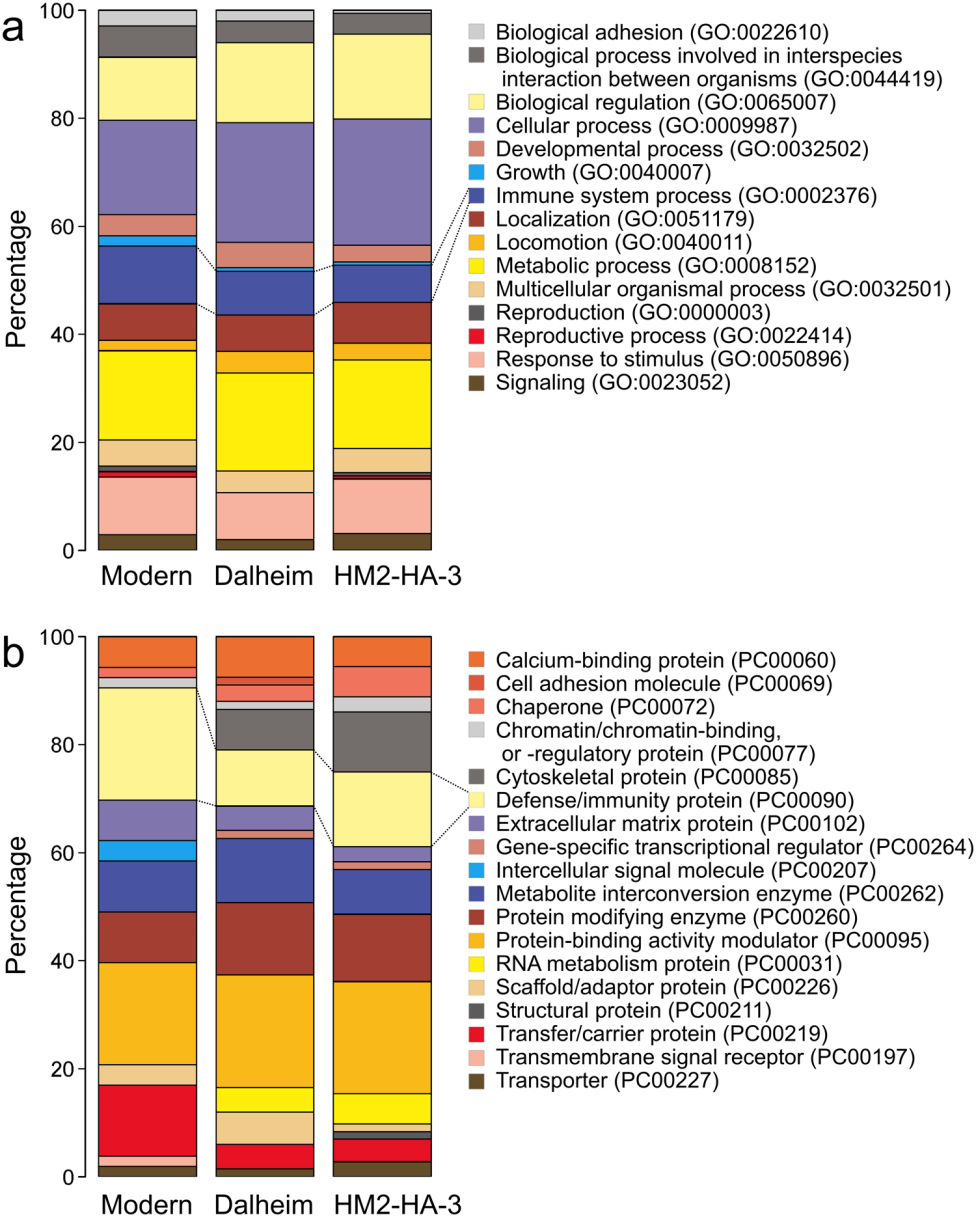
Results of PANTHER (**a**) biological process and (**b**) protein class analysis of protein groups identified in the dental calculus of HM2-HA-3 (this study) as well as in the dental calculus samples from medieval Dalheim and modern patients^12^.

The presence of various host defense response proteins suggests that HM2-HA-3 was subjected to pathological stress and the resulting inflammation, at least during dental calculus deposition. However, the identified host defense proteins were nonspecific (e.g., lactotransferrin, immunoglobulin kappa constant, and prolactin-inducible protein) and mostly similar to those identified in other ancient individuals with significantly lower calculus deposition (Supplementary Fig. S3)^13^. Moreover, our PANTHER analysis revealed that the “immune system process” comprised 6.9% of the total processes assigned to the identified host proteins in the HM2-HA-3 dental calculus (Fig. 4). This proportion is rather lower compared to those in the calculus samples from medieval Dalheim (8.1%) and modern patients suffering from moderate to moderate/severe periodontal disease (10.7%)^13^. Furthermore, the proportion of the “defense/immunity protein” class was also lower in the calculus of HM2-HA-3 (13.9%) than that in the modern dental calculus (20.8%) and was somewhat higher than that in the calculus sample of medieval Dalheim (10.4%)^13^. These results imply that host defense response to oral pathological stress was not necessarily higher in HM2-HA-3, who exhibited significant amounts of calculus deposits and severe masticatory dysfunction, relative to modern periodontitis patients and medieval individuals with lower calculus deposition.

Although palaeoproteomics provides molecular evidence on the bacterial etiology of and host defense response to periodontal disease, the cause of the abnormal calculus deposition in HM2-HA-3 remains unclear. Diet is often cited as a cause for calculus deposition^54^, but this cause is unlikely for HM2-HA-3. Stable isotope analysis showed that HM2-HA-3 had a similar diet to other individuals from the Hamanaka 2 site and other individuals from Hamanaka 2 site displayed little or no calculus deposition (Fig. 2). Abnormally high amounts of calculus deposition could occasionally be seen in modern patients, but the underlying cause is unidentifiable in most cases^55,56^. At least, this individual would not have a routine tooth cleaning habit during the period of calculus deposition. Furthermore, as the HM2-HA-3 bone proteome did not contain disease-indicative proteins, calculus deposition unlikely occurred as a systemic disease byproduct.

HM2-HA-3 is the first individual among the ancient human skeletons from Asia with a bacterial proteome studied in detail. Therefore, in this study, we used for comparison of previously published proteome results on calculi from individuals in Europe^12^. Almost all published bacterial proteomes of modern and ancient dental calculus originate from Europe^12,14^. As the regional differences in the human oral bacterial composition have been suggested^57^, accumulating data on dental calculus bacterial proteome outside Europe would be required.

## Materials and methods

Detailed procedures regarding sample collection and analyses are described in the Supplementary Materials and Methods. A brief summary is shown below.

### Sampling

Dental calculus was collected from the lower right first incisor of HM2-HA-3 (Supplementary Figure S1), with the method described previously^58^. Given the small variability in bacterial composition in calculus obtained from different oral positions within an individual^59^, we assume that this sample had a representative bacterial composition as would be obtained from the abnormally deposited calculus present on the molars (Fig. 1). Rib bones were also sampled for palaeoproteomic and isotope analyses.

### Proteomics

Protein extraction from 15 mg of dental calculus was performed using modified ultrafiltration and single-pot solid-phase-enhanced sample preparation (SP3) methods for ancient protein analysis^60,61^. Protein extraction from 20 mg rib bone was performed using modified ultrafiltration method^62^. Following the guidelines for palaeoproteomics^18^, the entire extraction process was carried out in a clean laboratory dedicated to ancient biomolecules built at the Graduate University for Advanced Studies, Japan. We obtained four fractions of the calculus sample (i.e., supernatant and pellet fractions from each of the ultrafiltration and SP3 methods) and two fractions (i.e., supernatant and pellet) of the bone sample along with experimental blanks.

Liquid chromatography-tandem mass spectrometry (LC–MS/MS) analysis of dental calculus was performed using an Orbitrap Fusion Tribrid mass spectrometer (Thermo Fisher Scientific) at Japan Agency for Marine-Earth Science and Technology (JAMSTEC) with the conditions described in Nunoura et al.^63^. LC–MS/MS analysis of rib bone was performed using an Orbitrap QE Plus mass spectrometer (Thermo Fisher Scientific) at Kanazawa University with the conditions described in Ogura et al.^64^. RAW data files generated by LC–MS/MS were analyzed using the MaxQuant software version 2.0.1.0^65^. Data of calculus were searched against the Oral Signature Screening Database (OSSD^20^) for the first quality-assurance step and the electric Human Oral Microbiome Database (eHOMD^66^) or entire human proteome (as of 2023-03-02) for the second protein identification step. Data of bone were searched against the entire human proteome. Because no food proteins were identified from dental calculus in a MaxQuant search against an entire Swiss-Prot database (as of 2021-08-20), we did not investigate food proteins. Comparative datasets were analyzed anew in the same manner^12^.

Gene Ontology (GO) analysis of the human-derived proteins identified from the dental calculus of HM2-HA-3 was performed using PANTHER, version 14^38^. Python script reported by Mackie et al.^14^ was used to calculate asparagine and glutamine deamidation rates. All subsequent data analyses were performed using R, version 4.2.2 (R Core Team, 2022).

### Radiocarbon dating and stable isotope analysis

Collagen was extracted from a rib bone of HM2-HA-3 to conduct radiocarbon measurement and carbon and nitrogen stable isotope analysis, based on the method described previously^67^. Carbon and nitrogen stable isotopes were measured using elemental analyzer-isotope ratio mass spectrometry (EA-IRMS) at the University Museum, the University of Tokyo (UMUT).

Radiocarbon concentrations were measured using accelerator mass spectrometry (AMS) at UMUT. Radiocarbon age was calibrated against atmospheric and marine calibration curves (IntCal20 and Marine20^68,69^) and with the local marine reservoir effect^70^ using OxCal, version 4.4^71^.

### Supplementary Information

Supplementary Information.

Supplementary Tables.

## Data Availability

LC–MS/MS data have been uploaded to PRIDE repository^72^ with the dataset identifier PXD044070.
